# Model-based prediction of myelosuppression and recovery based on frequent neutrophil monitoring

**DOI:** 10.1007/s00280-017-3366-x

**Published:** 2017-06-27

**Authors:** Ida Netterberg, Elisabet I. Nielsen, Lena E. Friberg, Mats O. Karlsson

**Affiliations:** Department of Pharmaceutical Biosciences, Uppsala University, Box 591, 751 24 Uppsala, Sweden

**Keywords:** Self-monitoring of ANC, Model-based predictions, Chemotherapy-induced myelosuppression, Docetaxel

## Abstract

**Purpose:**

To investigate whether a more frequent monitoring of the absolute neutrophil counts (ANC) during myelosuppressive chemotherapy, together with model-based predictions, can improve therapy management, compared to the limited clinical monitoring typically applied today.

**Methods:**

Daily ANC in chemotherapy-treated cancer patients were simulated from a previously published population model describing docetaxel-induced myelosuppression. The simulated values were used to generate predictions of the individual ANC time-courses, given the myelosuppression model. The accuracy of the predicted ANC was evaluated under a range of conditions with reduced amount of ANC measurements.

**Results:**

The predictions were most accurate when more data were available for generating the predictions and when making short forecasts. The inaccuracy of ANC predictions was highest around nadir, although a high sensitivity (≥90%) was demonstrated to forecast Grade 4 neutropenia before it occurred. The time for a patient to recover to baseline could be well forecasted 6 days (±1 day) before the typical value occurred on day 17.

**Conclusions:**

Daily monitoring of the ANC, together with model-based predictions, could improve anticancer drug treatment by identifying patients at risk for severe neutropenia and predicting when the next cycle could be initiated.

**Electronic supplementary material:**

The online version of this article (doi:10.1007/s00280-017-3366-x) contains supplementary material, which is available to authorized users.

## Introduction

The dose-limiting toxicity for most cytotoxic chemotherapeutic drugs in use today is bone-marrow toxicity [1]. However, some level of hematologic toxicity is warranted since moderate neutropenia has been associated with improved survival [2–4]. A low degree of toxicity may be a sign of low exposure, and consequently sub-optimal effect. Docetaxel is a commonly used anti-cancer agent [5–7] that possess pronounced myelosuppressive properties [8], which exposes the patient to a high risk for severe neutropenia and consequently life-threatening conditions such as febrile neutropenia (FN). Docetaxel, typically administered as a 1 h infusion every 3 weeks in multiple cycles depending on the cancer type, can cause high rates (>20%) of FN [9]. Prior to each administration of cytotoxic drugs, the absolute neutrophil count (ANC) is measured to confirm adequate levels, i.e. ANC ≥ 1.5 × 10^9^ cells/L for docetaxel (Taxotere^®^ label). At some institutions the ANC is also routinely monitored at the expected nadir, i.e. around day 7 for docetaxel. Potential limitations of a sparse ANC monitoring include (1) delay in identification of patients at high risk of experiencing life-threatening conditions, (2) inconvenience for the patient and the clinic if the next cycle needs to be delayed due to low ANC [10, 11] the same day the patient is scheduled for the next cycle, and (3) too cautious choice of dose and thereby suboptimal therapy.

Frequent monitoring has recently become a realistic possibility with the development of a self-testing device that patients can use to measure their white cell count at home, without additional medical assistance [12]. Clinicians can thereby follow the patient’s ANC by a remote access of the data. The addition of real-time model-based predictions of such data could further contribute to therapeutic options such as: (1) prediction of time and depth of nadir, i.e. lowest ANC, before it is reached, (2) prediction of a suitable time for the next sample, (3) predictions for whether the patient will have recovered sufficiently in immuno-competence to receive the next course on the planned date, and (4) the expected consequence of planned dose(s) for coming schedules. In this work, we outline some basic properties of the expected predictive performance based on frequent neutrophil measurements and a population model of myelosuppression.

Models in oncology can quantify the pharmacokinetics of anti-cancer drugs as well as the pharmacodynamic effect, e.g. survival or side effects such as hematological toxicity, and how the different effects relate to each other [13]. In 2002, a semi-mechanistic model describing the loss and recovery of leukocyte and neutrophil counts after exposure to myelosuppressive chemotherapy was proposed [14]. This model was found to be applicable across several drugs with relatively few parameters needed to be estimated. The following mechanistic aspects of chemotherapy-induced myelosuppression was included in the model: (1) a self-renewal mechanism, regulating a compartment of proliferative cells, (2) a feedback parameter that increases the production when the number of blood cells is reduced, e.g. mimicking the effects of granulocyte-colony stimulating factor (G-CSF), (3) a cell maturation process to describe the time-delay between effects on production and changes in circulatory neutrophil counts and (4) a distinct differentiation between system-related and drug-related parameters. Since the proposal of this semi-mechanistic model, it has been used in numerous settings. Multiple variables have been evaluated to improve the predictive properties of the model [15–17]. The model has also been used to design and evaluate dosing schedules in drug development [18–21], to explore its potential for individualized feedback-adaptive dosing in the clinic [22] and to incorporate measured G-CSF concentrations [23].

In this study we first simulated daily ANC based on the myelosuppression model after docetaxel administration and in a subsequent step used the same model to predict ANC later in the cycle based on different amount of the neutrophil data. The purpose was to investigate if and how a more frequent monitoring of the ANC together with model-based analysis could improve the management of patients on myelosuppressive therapy.

## Materials and methods

Daily ANC values were simulated for 600 patients from the earlier described myelosuppression model [14, 16] where the time-course of docetaxel-induced neutropenia had been characterized. The “true” individual profiles for the myelosuppression time-course were defined from the individual parameters used to simulate the data. Subsequently, varying numbers of the simulated ANC values (with respect to monitoring duration and frequency) were used to predict individual profiles for the myelosuppression time-course. Forecasts of the ANC were made, comparing the true to the predicted ANC, and the accuracy of the forecasts was assessed. Summary variables, i.e. (1) the time to nadir, NADIR_time_, (2) the ANC value at nadir, NADIR_ANC_, and (3) the time for the ANC to recover to the baseline ANC value, RECOVERY-ANC0_time_, were also computed and the imprecision of these predictions was evaluated. Lastly, sensitivities and specificities were calculated for Grade 4 neutropenia and ANC ≤ 0.1 × 10^9^ cells/L. Docetaxel was used as an example drug in this analysis since it causes a high frequency of severe neutropenia and febrile neutropenia. It may therefore be of interest to monitor the ANC for patients receiving docetaxel. The different parts are described more in detail below.

### Simulations of the ANC

**Fig. 1:**
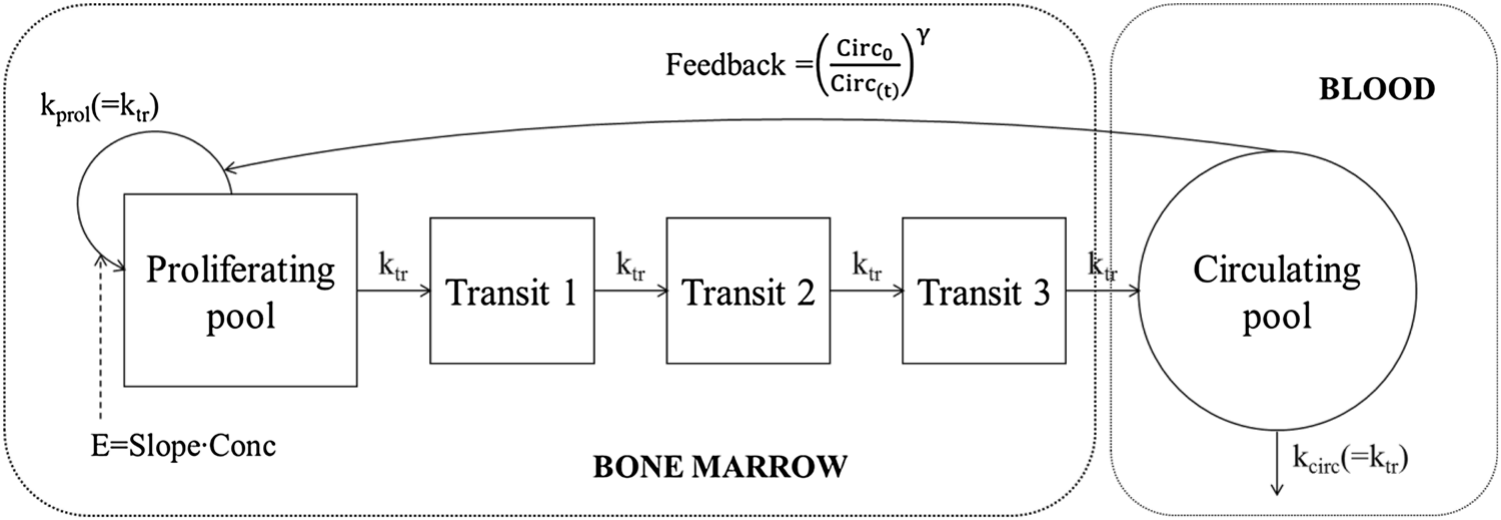
A schematic representation of the myelosuppression model, originally published by Friberg et al. E is the linear drug effect, mediated through the Slope parameter and the drug concentration (Conc). *k*_tr_ is the transition rate constant, *k*_prol_ is the proliferation rate constant and *k*_circ_ is the elimination rate constant. Circ_0_ is the baseline absolute neutrophil count (ANC) and Circ_(*t*)_ is the ANC in the circulating pool at time *t*. *γ* is the parameter regulating the feedback system between cell proliferation and cell elimination

Individual predicted docetaxel concentration time-profiles were available from a previous population pharmacokinetic analysis, where docetaxel was given alone as a 1-h infusion of 75 or 100 mg/m^2^ every 3 weeks [16]. The myelosuppression model by Friberg et al. [14] (Fig. 1) was here applied with ANC parameter estimates according to Kloft et al. [16] (Table 1) with the exception that in the current analysis no variability in the feedback parameter γ was included [24]. It was decided to apply the parameter estimates from Kloft et al. since this model was developed on logarithmic data, in contrast to the model by Friberg et al., which has been recognized as superior for neutrophil data to capture the nadir. Also, the model by Kloft et al. is an updated version of the myelosuppression model including parameter-covariate relationships. Given the model and its parameters, the ANC was simulated just before the start of docetaxel treatment (baseline) and thereafter daily from day 3 to day 21 (first cycle only) for 600 patients with the same predicted docetaxel concentrations, demographics and patient characteristics as in the original data set [16]. The difference between the “true” ANC and the simulated ANC is the residual error that for example includes measurement error. No ANC values were simulated on day 1 or 2 since glucocorticoids are often administered before and/or early in the cycle [25], leading to a short temporal increase in circulating neutrophils [26, 27], which is not captured by the model.

**Table 1:** Parameter estimates used to simulate and predict from the myelosuppression model

Parameter	Estimate (95% CI^a^)	IIV^b^, CV^c^ % (95% CI)
ANC_0_^d^ (10^9^ cells/L)	5.22 (4.93–5.51)	25 (22–28)
MTT^e^ (h)	84.2 (82.7–85.7)	14 (13–15)
*γ* ^f^	0.145 (0.141–0.149)	0 FIX
Slope (L/μmol)	15.6 (15.1–16.2)	36 (33–39)
Residual error (%)	42.4 (39.6–45.2)	
Parameter-covariate relationships		
ANC_0_–AAG^g^		
If AAG ≤ 1.34	0.175 (0.130–0.220)	
If AAG > 1.34	0.495 (0.262–0.728)	
ANC_0_-sex^h^	−0.121 (−0.176 to −0.066)	
ANC_0_-performance status^i^	0.131 (0.067–0.195)	
ANC_0_-previous chemotherapy^j^	−0.147 (−0.202 to −0.092)	
Slope-AAG^k^	−0.351 (−0.363 to −0.339)	

These estimates were generated in the analysis by Kloft et al., with the exception that the IIV related to the feedback parameter, *γ*, was set to 0 in the current analysis

The distribution [median (range)] of the continuous covariates were as follows: age: 56 (26–80); AAG: 1.34 g/L (0.29–3.57)

The distribution (proportions) of the categorical covariates were as follows: sex: 43% male, 57% female; performance status: 34% 0 or unknown, 66% ≥1; previous chemotherapy: 44% yes, 56% no

^a^Confidence interval

^b^Interindividual variability

^c^Coefficient of variation

^d^Baseline ANC

^e^Mean transit time

^f^Feedback parameter

^g^Alpha 1-acid glycoprotein

^h^Categorical relationship, lower ANC_0_ for females

^i^Categorical relationship, higher ANC_0_ for patients with higher performance status

^j^Categorical relationship, lower ANC_0_ for patients who had previous chemotherapy

^k^Linear relationship

Since docetaxel is known to frequently cause myelotoxicity, the same procedure as described above was applied to a hypothetical drug with less myelosuppressive properties. This was done by simulating from the same model but the toxicity parameter “Slope” was set to half of its original value. Also, the impact of a smaller residual error (i.e. approximately 26%, which is the same as for paclitaxel in the analysis by Kloft et al.) and a longer mean transit time (MTT) (i.e. 141 h, which was the longest estimated MTT in the analysis by Kloft et al.), were also explored.

### Predictions of the ANC

**Fig. 2:**
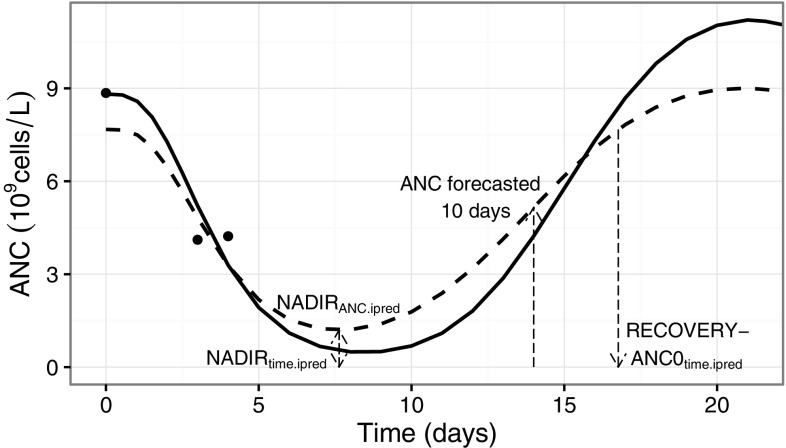
The absolute neutrophil count (ANC) versus time during chemotherapy-induced myelosuppression. The *solid* and *dashed lines* represent the true and predicted time-courses, respectively. The *dots* represent the simulated data that was used to generate the predicted profile in one scenario (ANC only available at baseline and day 3 and 4). The *arrows* (*long-dashed lines*) indicate the predicted nadir time (NADIR_time.ipred_) nadir ANC (NADIR_ANC.ipred_) and time to baseline recovery (RECOVERY-ANC0_time.ipred_). A 10-day forecast of the ANC from the time of last observation is also indicated by an *arrow* in the figure

A Bayesian feedback analysis was performed, i.e. no (re-)estimation of population parameters but the simulated data and the original model population parameter estimates (Table 1) were used to estimate individual parameters and predict individual ANC profiles. This method has been described previously in the pharmacometric setting [28]. Predicted individual myelosuppression time-course profiles, referred to as ANC_ipred_, were obtained from a range of scenarios with different amount of ANC measurements, given the same model as used for the simulations. The evaluated scenarios varied in number of measurements available based on (1) monitoring frequency (daily, every other and every third day) and (2) monitoring duration, i.e. data available up to a given day in the cycle. Two additional scenarios, annotated as the baseline and baseline and day 5 scenarios, were also included. Only one measurement at baseline was available in the baseline scenario and for the baseline and day 5 scenario, a measurement at baseline and day 5 were available. The varying amount of data was used to investigate the properties of prospective predictions. Based on the simulated data, the ANC was predicted later on in the cycle. Figure 2 illustrates a scenario where the ANC was monitored at baseline and then at day 3 and 4 and predictions were made based on that data only. The true profile is included in the figure to clarify the difference between the true and predicted profiles.

Forecasts of the ANC (exemplified in Fig. 2) were made for the different scenarios. The accuracy for each of the forecasts was evaluated as an absolute error on the log-scale (simulated and predicted data were on log-scale), Eq. 1, for each individual in the data set.

1 $${\text{Error}}_{d,D} = \log \left( {{\text{ANC}}_{{{\text{ipred}}.,d,D}} } \right) - { \log }({\text{ANC}}_{{{\text{true}},D}} )$$

ANC_ipred.,*d*,*D*_ is the individual predicted ANC at day *D*, given data available up to day *d* and ANC_true,*D*_ is the true ANC at day *D*, defined as the simulated individual value without residual error.

### Summary variables of the myelosuppression time-course profile

The summary variables of the myelosuppression time-course profile, i.e. NADIR_time_, NADIR_ANC_ and RECOVERY-ANC0_time_ (illustrated in Fig. 2), were predicted by Bayesian feedback using the myelosuppression model and the available data in each scenario. The values obtained from the true and predicted profiles were compared and evaluated with respect to both bias and imprecision as a root-mean squared error on day *d*, RMSE_*d*_. The RMSE_*d*_ was calculated both in the actual unit of the variable and as a percent, Eqs. 2 and 3, respectively,

2 $${\text{RMSE}}_{d} = \sqrt {\frac{{\mathop \sum \nolimits_{i = 1}^{N} \left( {{\text{Value}}_{{{\text{ipred}},d}} - {\text{Value}}_{{{\text{true}},i}} } \right)^{2} }}{N}}$$

3 $${\text{RMSE}}\%_{d} = \sqrt {\frac{{\mathop \sum \nolimits_{i = 1}^{N} \left( {\frac{{{\text{Value}}_{{{\text{ipred}},d}} - {\text{Value}}_{{{\text{true}},i}} }}{{{\text{Value}}_{{{\text{true}},i}} }}} \right)^{2} }}{N}}$$

Value_ipred,*d*_ is the individual predicted value of the summary variable, given data available up to day *d*. Value_true,*i*_ is the true value of the summary variable for the *i*th individual. *N* is the number of patients in the data set.

### Occurrence of severe neutropenia

From the true ANC time-course, patients were classified in two ways with different cut-offs for severity of the neutropenia; first as experiencing or not experiencing Grade 4 neutropenia (ANC < 0.5 × 10^9^ cells/L) and secondly as having or not having an ANC ≤ 0.1 × 10^9^ cells/L. The same classifications were made for the predicted time-courses. By comparing the true and predicted classifications, sensitivities and specificities were computed for the different scenarios.

### Data analysis

The analysis was performed using a software for non-linear mixed effects models, NONMEM 7.2 and 7.3 [29]. Perl speaks NONMEM (PsN) and Pirana was used to execute the simulations and the predictions in NONMEM and to facilitate documentation of a run record, respectively [30]. The accuracy, bias and imprecision were computed in R version 3.2 (www.R-project.org). R, together with the graphical visualization package ggplot2 [31], was used for producing the figures.

## Results

### Predictions of the ANC

**Fig. 3:**
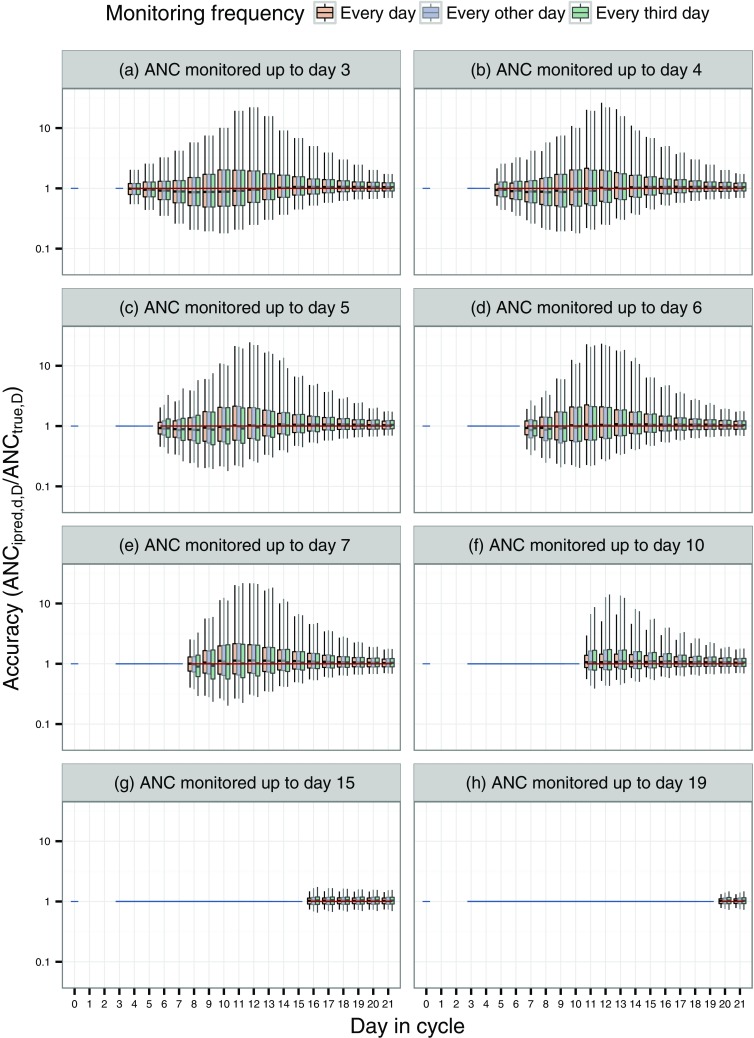
Distribution of the absolute error for scenarios where the ANC was monitored until day 3, 4, 5, 6, 7, 10, 15 and 19. ANC_ipred.,*d*,*D*_ is the individual predicted ANC at day *D*, given data available up to day *d* and ANC_true,*D*_ is the true ANC at day *D*. *Orange*, *blue* and *green boxes* represent monitoring frequency every, every other and every third day. The *horizontal line* represents no prediction error, the *blue* and *red lines* illustrate days the ANC was monitored and predicted, respectively. The *vertical line* inside of each box is the median. *Lower* and *upper hinges* of the *box* represent the 25th and 75th percentiles, respectively. *Lower* and *upper ends* of the *whiskers* correspond to the 2.5th and 97.5th percentiles, respectively

The distributions of the accuracy (ANC_ipred._/ANC_true_, where ANC_ipred._ is the predicted ANC for each subject in each scenario and ANC_true_ is defined from the individual parameters used to simulate the data) in the forecasts for the 600 simulated subjects are presented in Fig. 3 for a selected number of monitoring days. The widest distributions were observed for forecasts around the time of nadir, e.g. the 2.5th and 97.5th percentiles of the accuracy values were 0.27 and 20.2 for a 4-day forecast with daily data monitored up to day 7 (the fourth orange box in plot e in Fig. 3). The distribution of the accuracy values reduced for days later in the cycle and as more data became available for the predictions. When ANC was monitored beyond the time of nadir, the forecasts became more certain, e.g. the 2.5th and 97.5th percentiles of the accuracy values were 0.74 and 1.43, respectively, when the ANC was monitored up to day 15 and predicted at day 21 (sixth white box in plot g in Fig. 3). Generally, the spread of the error was lower with daily monitoring, compared to monitoring every other or every third day, although the differences were modest.

For the predictions based on the smaller “Slope” value, mimicking a drug with a less profound myelosuppressive effect, narrower distributions of the accuracy were observed (Fig. 1 in Online Resource 1). The impact of the smaller residual error was negligible (Fig. 1 in Online Resource 2). The accuracy was better than for docetaxel around nadir, when a longer MTT was used. Although, the accuracy was as expected lower than for docetaxel for later times in the cycle (Fig. 1 in Online Resource 3).

### Summary variables of the myelosuppression time-course profile

**Fig. 4:**
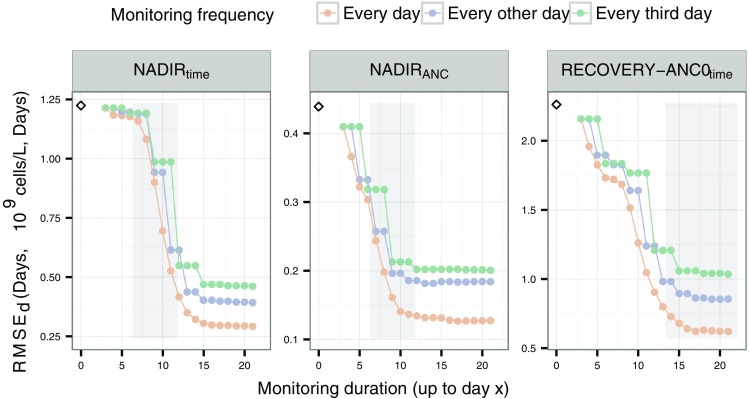
Root-mean squared error at day *d* in the cycle (RMSE_*d*_) of NADIR_time_, NADIR_ANC_ and RECOVERY-ANC0_time_. The *dots* represent the errors, connected by *lines*. *Orange*, *blue* and *green colors* indicate the daily, every other and every third day monitoring of the ANC, respectively. The *empty diamond* represents the RMSE_*d*_ of the scenario with data available only at baseline. The *shaded grey areas* represent 95% (2.5th to 97.5th percentiles) of the true times of nadir and recovery to baseline, respectively

The influence on precision and bias of the predictions of NADIR_time_, NADIR_ANC_ and RECOVERY-ANC0_time_, with respect to ANC monitoring frequency and monitoring duration is illustrated in Fig. 4 (results based on the smaller “Slope” value, smaller residual error and longer MTT are given in Fig. 2 in Online Resource 1, 2 and 3, respectively). It is worth mentioning that the precision and bias were larger when the longer MTT was used. A possible explanation is the size of the ANC around nadir (0.26 × 10^9^ cells/L for docetaxel and 0.81 × 10^9^ cells/L for the longer MTT, true median values). The predictions of NADIR_time_, NADIR_ANC_ and RECOVERY-ANC0_time_ showed the least imprecision, i.e. a low RMSE_d_, in scenarios with daily monitoring of the ANC and the more extended monitoring durations, similar to the results for the ANC forecasts. It was found that RECOVERY-ANC0_time_ could be predicted with an imprecision (RMSE_*d*_) of ±1 day with daily monitoring of the ANC and a monitoring duration of 11 days after the docetaxel administration (left plot in Fig. 4). The true median RECOVERY-ANC0_time_ was approximately 17 days and no improvement in the RMSE_*d*_ of RECOVERY-ANC0_time_ was observed in scenarios with longer monitoring durations than 17 days. In contrast to RECOVERY-ANC0_time_, the RMSE_*d*_ of NADIR_time_ and NADIR_ANC_ continued to improve in scenarios with longer ANC monitoring durations than the true median NADIR_time_. In the scenario with daily ANC monitoring and a monitoring duration of roughly 1 day shorter than the true median NADIR_time_, the RMSE_*d*_ of NADIR_time_ and NADIR_ANC_ was more than ±1 day and ±0.2 × 10^9^ cells/L, respectively.

In Fig. 4 the RMSE_*d*_ of the baseline scenario, together with the RMSE_*d*_ in scenarios with daily monitoring of the ANC, is presented. In terms of bias and precision, a single measurement at baseline performs worse for all three variables compared to having additional measurements. The RMSE_*d*_ (RMSE_*d*_ %) of NADIR_time_, NADIR_ANC_ and RECOVERY-ANC0_time_ decreased from 1.22 days (13.6%), 0.439 × 10^9^ cells/L (805%) and 2.26 days (12.9%) in the scenario where only a baseline measurement was available, to 1.18 days (12.8%), 0.303 × 10^9^ cells/L (346%) and 1.73 days (9.77%) in the scenario with daily ANC monitoring and a monitoring of the ANC until day 6, i.e. before occurrence of the true median NADIR_time_.

### Occurrence of severe neutropenia

**Fig. 5:**
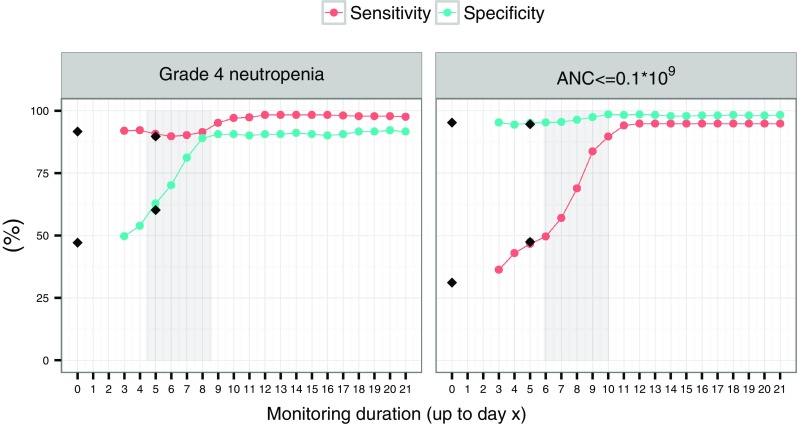
The *dots* represent the sensitivity and specificity for classification of Grade 4 neutropenia (*left*) and an ANC ≤ 0.1 × 10^9^ cells/L (*right*), based on daily monitoring of the ANC, connected by *lines*. The *diamonds* represent the baseline and baseline and day 5 scenarios. The *shaded grey areas* represent 95% (2.5th to 97.5th percentiles) of the true times for occurrence of Grade 4 neutropenia (*left*) and an ANC ≤ 0.1 × 10^9^ cells/L (*right*)

Given the true individual profiles, 68 and 23% of the patients in the simulated data set experienced Grade 4 neutropenia and an ANC ≤ 0.1 × 10^9^ cells/L, respectively, on at least one day during the simulated treatment cycle. The median time for the first day of Grade 4 neutropenia and an ANC ≤ 0.1 × 10^9^ cells/L in the simulated data set was at day 6.0 and 7.6 in the cycle, respectively. The results for daily monitoring are illustrated in Fig. 5 (results based on the smaller “Slope” value, smaller residual error and longer MTT are given in Fig. 3 in Online Resource 1, 2 and 3, respectively). For Grade 4 neutropenia the sensitivity was high, even in the baseline scenario (91.9%). On the contrary, the specificity was clearly lower in the baseline scenario (48.7%). The opposite pattern was observed for the more severe neutropenic case where the ANC was ≤0.1 × 10^9^ cells/L (30.4 and 94.8% for the sensitivity and specificity, respectively, in the baseline scenario). Mainly the Grade 4 neutropenia specificity and ANC ≤ 0.1 × 10^9^ cells/L sensitivity improved with longer monitoring durations. The difference in sensitivity and specificity for the baseline + day 5 scenario and the scenario when the ANC was monitored at baseline + day 3, 4 and 5 was negligible.

When the predictions were based on the smaller “Slope” value the sensitivity and specificity patterns for Grade 4 neutropenia and ANC ≤ 0.1 × 10^9^ cells/L were different. The sensitivities were lower for the baseline scenario for both Grade 4 neutropenia and ANC ≤ 0.1 × 10^9^ cells/L (Fig. 3 in Online Resource 1). On the other hand, the specificities were higher for both cut-offs, i.e. close to 100%. The reason is most likely the differences in frequency of severe neutropenia in the simulated data sets, typically 68% (original “Slope” value) vs 19% (half of the original “Slope” value) for Grade 4 neutropenia and 23% (original “Slope” value) vs 2.8% (half of the original “Slope” value) for ANC ≤ 0.1 × 10^9^ cells/L.

## Discussion

Frequent self-monitoring of neutrophil counts has potential for improving the care of patients undergoing myelosuppressive therapy. As mentioned in the introduction, limited ANC monitoring can have an impact on the treatment in a number of ways. For example, most often it is not observed until the patient arrives at the clinic for the next dose administration that the ANC has not recovered to a sufficiently high value and this will have the consequence that the dose will be needed to be delayed and the patient has to return on a later day [10, 11]. Delaying the cycle could also affect the patient mentally [32] and cause unnecessary stress. Postponed treatment cycles may also lead to inconvenience for the clinic, administratively and/or financially. For convenience and in order to fit the clinics’ schedules, the cycle is typically delayed one week. Postponing the initiation of the subsequent cycle(s) has been related to worse disease-free and overall survival [33]. If the ANC could be forecasted a few days in advance, it would be possible for the clinic to individualize the day of dose administration instead of postponing for a longer period than would be needed for ANC recovery.

The myelosuppression model by Friberg et al. [14] with parameter estimates according to Kloft et al. [16]. was here applied to explore how well the ANC, and summary variables of its time-course, can be predicted with varying amount of data available for making the predictions, using docetaxel as an example drug. The objective of this study was not to characterize the quality or robustness of the model itself, but to illustrate the potential and limitations of using frequent ANC monitoring together with model-based predictions to improve patient management. The model by Kloft et al. was used in the current analysis since the modelled ANC was log-transformed and captured the nadir better than the model by Friberg et al., where the data was not transformed. The myelosuppression model describes several physiological aspects of chemotherapy-induced myelosuppression and has therefore shown to be valuable to facilitate different aspects of drug development [21] and for dose-individualized feedback adaption [22]. It should, however, be noted that in this analysis it is assumed that the model is true, and that this study therefore represents a “best-case-scenario”.

As expected, the predictions were more accurate and precise when more measurements were available from the patient and when the forecast was short. By monitoring the ANC, predictions of future ANC values will be more certain than using a single measurement at baseline. However, the predictions of the exact value of ANC at and around nadir were associated with uncertainty; the RMSE_*d*_ of the predictions of NADIR_ANC_ were large when only a few ANC measurements were available. NADIR_time_ was neither very well predicted. It is however expected to observe some uncertainty of NADIR_ANC_ and NADIR_time_, due to the high residual error (42%) [16]. On the contrary, the sensitivity to identify Grade 4 neutropenia was relatively high, even for the baseline scenario. Therefore, predictions of the risk for a patient to develop Grade 4 neutropenia could help identifying patients that might be in need of rescue-medication, e.g. growth factor such as G-CSF. According to several guidelines [34–37], G-CSF may also be used as primary prophylaxis if the risk for FN is ≥20%, based on age, medical history, disease characteristics and myelotoxicity of the chemotherapy regimen [36]. G-CSF is also recommended for all subsequent cycles as a secondary prophylaxis if the patient had an FN episode in a previous cycle [34]. FN is a severe condition and patients with Grade 4 neutropenia who develop fever, i.e. FN, may face life-threatening infections that need to be treated with antibiotics [38, 39]. The overall mortality for patients with FN and solid and hematological malignancies is ~5 and 11%, respectively [38]. The risk for the individual patient already diagnosed with FN to develop severe complications is very much dependent on patient factors.

From the results in this study it was shown that it is possible to predict, several days before the planned start of the next cycle, if the following dose could be administered on the planned day or not (Fig. 3). Docetaxel is contraindicated if the pretreatment ANC is <1.5 × 10^9^ cells/L and it can therefore be of interest to predict when a patient will recover above this threshold or to its baseline ANC and thereby guide when it can be a good time to schedule the next dose administration. RECOVERY-ANC0_time_ could be predicted ±1 day 6 days before the true median time it takes for a patient to recover to baseline ANC (Fig. 4), i.e. around day 11. These results indicate that for many patients, the subsequent cycle could be initiated even earlier than after 21 days after last dose, for the studied regimen. A 21-day cycle is, however, partly applied for practical reasons and it should be acknowledged that individualizing the time for dose delivery could be associated with an additional work load for the clinic. An additional concern for administering the subsequent dose earlier than after 21 days after the last dose, is the activity of the bone-marrow. Even if the ANC in blood has recovered enough for the next dose to be given, the bone-marrow might be in an active phase where it is still in a highly proliferating stage and therefore extra sensitive for the effect of cytotoxic drugs. These results would therefore be of interest to combine with studies on the effect of individualized administration time on survival and safety, as well as its impact on healthcare resources and patient convenience.

Docetaxel, which is known to cause high rates of severe neutropenia, was used as an example drug in this work. The approach, i.e. frequent ANC monitoring together with model-based predictions, can of course be extended to other regimens and anti-cancer therapies where neutropenia may be a concern. However, the results in this study may not reflect the accuracy of the predictions for another therapy. As an extension to the predictions based on docetaxel, simulations and predictions were performed where the parameter describing the cytotoxic effect (“Slope”) was set to half of its original value. It was shown that the forecasts were less variable and that the sensitivity for predicting Grade 4 neutropenia and ANC ≤ 0.1 × 10^9^ cells/L was lower. Predictions based on a smaller residual error showed no major differences compared to the estimated docetaxel residual error in the Kloft analysis. A longer MTT resulted in both higher and lower accuracy and precision, probably because the larger size of the ANC at nadir, which also occurs later in the cycle.

In the study presented here, simulated daily ANC measurements were used to investigate the objective. Although it is not reasonable that the ANC would be assayed by health care professionals on a daily basis, recently technical advances [12] allow for patients to measure their ANC by themselves at home. If the clinician get access to the ANC measurement and, for example, temperature measurements in real-time he/she can make a professional assessment whether or not the patient is at risk for severe neutropenia and/or FN. A natural extension to this process would be to include model-predictions of expected ANC.

In this analysis, the frequency of the ANC monitoring was explored for daily, every other and every third day monitoring. Docetaxel was used as an example drug. Depending of the variable that is desired to predict, it might not be necessary for the patient to measure the ANC every day. As illustrated in Fig. 3, there was only a small difference in the accuracy for the daily, every other and every third day monitoring. It could be enough to measure the ANC only every third day, if the objective is to predict what the ANC will be on day 21. An additional option could be to monitor the ANC with varying frequency over the cycle, e.g. more frequently around nadir and then more sparsely later on in the cycle when the ANC is recovering and additional measurements add less information.

In conclusion, limited ANC monitoring may cause inconvenience both for the patient and the clinic as well as suboptimal therapy. With home-based labs, more frequent monitoring will be possible and together with model-based predictions the day of initiation of the subsequent cycle can be individualized and patients at high risk for severe neutropenia can be identified.

## Electronic supplementary material

Below is the link to the electronic supplementary material.

Supplementary material 1 (DOCX 321 kb)

Supplementary material 2 (DOCX 330 kb)

Supplementary material 3 (DOCX 327 kb)
