# Supplementary material for: Model-based prediction of myelosuppression and recovery based on frequent neutrophil monitoring
Source: Cancer Chemother Pharmacol. 2017 Jun 27;80(2):343–53. doi: 10.1007/s00280-017-3366-x (PMC5532422; doi:10.1007/s00280-017-3366-x)
Supplement: Supplementary file 1 — Supplementary material 1 (DOCX 321 kb) [file 280_2017_3366_MOESM1_ESM.docx]

# Online Resource 1

##
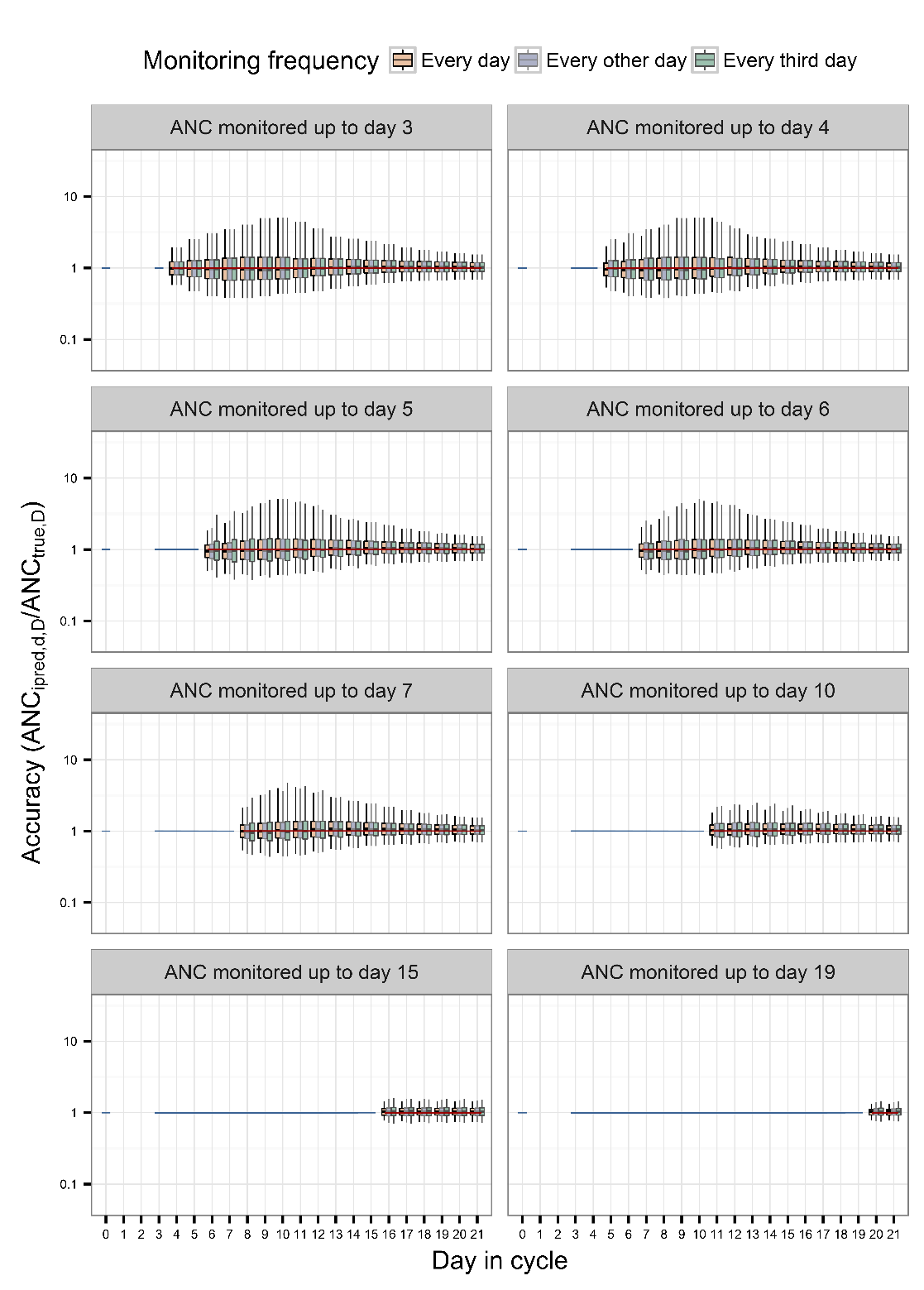


**Fig. 1** Distribution of the absolute error for scenarios where the ANC was monitored until day 3, 4, 5, 6, 7, 10, 15 and 19. ANC_ipred.,d,D_ is the individual predicted ANC at day *D*, given data available up to day *d* and ANC_true,D_ is the true ANC at day *D*. Orange, blue and green boxes represent monitoring frequency every, every other and every third day. The horizontal line represents no prediction error, the blue and red lines illustrate days the ANC was monitored and predicted, respectively. The vertical line inside of each box is the median. Lower and upper hinges of the box represent the 25^th^ and 75^th^ percentiles, respectively. Lower and upper ends of the whiskers correspond to the 2.5^th^ and 97.5^th^ percentiles, respectively. This figure illustrates results based on when the “Slope” parameter was set to half of its original value.


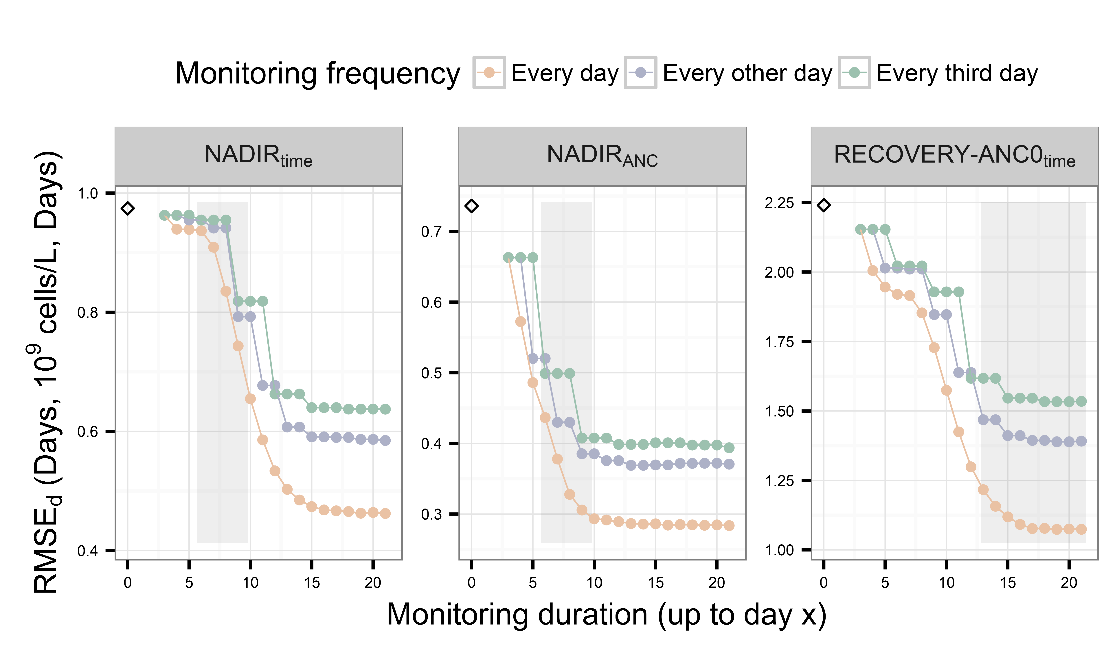


**Fig. 2** Root-mean squared error at day *d* in the cycle (RMSE_d_) of NADIR_time_, NADIR_ANC_ and RECOVERY-ANC0_time_. The dots represent the errors, connected by lines. Orange, blue and green colors indicate the daily, every other and every third day monitoring of the ANC, respectively. The empty diamond represents the RMSE_d_ of the scenario with data available only at baseline. The shaded grey areas represent the 95% confidence interval of the true times of nadir and recovery to baseline, respectively. This figure illustrates results based on when the “Slope” parameter was set to half of its original value.


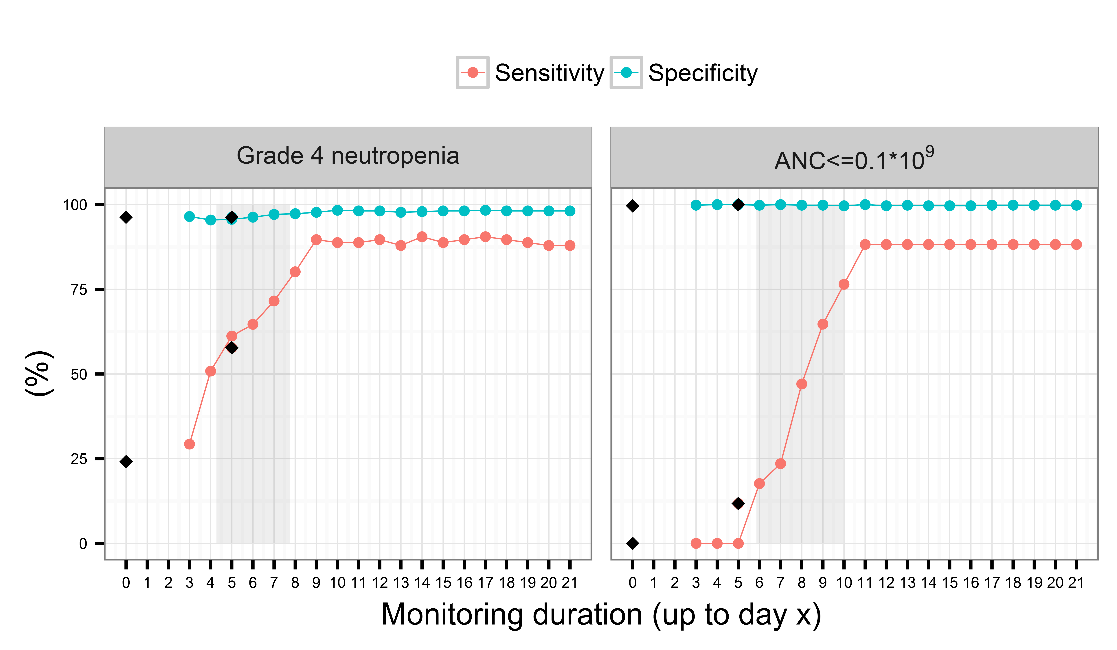


**Fig 3.** The dots represent the sensitivity and specificity for classification of Grade 4 neutropenia (left) and an ANC≤0.1·10^9^ cells/L (right), based on daily monitoring of the ANC, connected by lines. The diamonds represent the baseline and baseline and day 5 scenarios. The shaded grey areas represent the 95% confidence interval of the true times for occurrence of Grade 4 neutropenia (left) and an ANC≤0.1·10^9^ cells/L (right). This figure illustrates results based on when the “Slope” parameter was set to half of its original value.

**Model-based prediction of myelosuppression and recovery based on frequent neutrophil monitoring**

Cancer Chemotherapy and Pharmacology

Ida Netterberg^1^, Elisabet I. Nielsen^1^, Lena E. Friberg^1^, Mats O. Karlsson^1,2^

^1^Department of Pharmaceutical Biosciences, Uppsala University, Uppsala, Sweden

^2^Corresponding author:

Mats Karlsson, PhD

E-mail: [mats.karlsson@farmbio.uu.se](mailto:mats.karlsson@farmbio.uu.se)
